# The School Clinic: Diseases of the Eye

**Published:** 1912-02-10

**Authors:** 


### THE SCHOOL CLINIC: Diseases of the Eye.

The children referred by the school inspector for defects of vision practically all need spectacles. There are various societies in existence for the supply of these articles to the children, and it is the duty of the State not to waste the money of the ratepayers or the charitable by putting the work in the hands of men incapable of correctly rectifying the errors of refraction.

Of all the branches of specialism which have evolved during the past quarter of a century none is so special as the estimation of errors of refraction, and these errors are most difficult correctly to estimate in the case of children. It needs constant practice both to learn and to maintain. To say that a man who has done three months' dressership in an ophthalmic clinic is capable of correctly estimating such errors of refraction is as absurd as to say that the newly qualified man who has answered one question on public health in his final examination is capable of looking after the sanitary arrangements of a new block of buildings. And it is as absurd to put a school clinic dealing with refractions in the hands of the one as to ask the other to superintend plumbing. All school clinics dealing with errors of refraction should be in the hands of men with very considerable experience in their estimation; they might have under them others who are as yet learning the work, since the supply could only be kept up by teaching more men; but those who are learning should be able to give up three mornings a week. We would as soon see such clinics in the hands of opticians who gave their entire time to the work as in the hands of medical men who had not had a prolonged training in it.

The organisation of eye clinics differs from that of throat or dental clinics in that the children do not need to attend more than twice, and therefore one clinic would suffice to serve a larger area and a larger number of schools. In this way the number of very specialised refractionists would be diminished and the distribution of spectacles could be carried out more economically. Again, economy would be maintained by saving the money that must be expended if sets of lenses had to be procured for a large number of smaller clinics.

